# Validating a T1-weighted cine MRI for a 1.5T MR-Linac with temporal resolution appropriate for respiratory motion

**DOI:** 10.3389/fonc.2025.1575001

**Published:** 2025-06-04

**Authors:** Nathan Shaffer, Alex Dresner, Qi Ying, Eveline Alberts, Marijn Kruiskamp, Joseph Caster, Daniel Hyer, Jeffrey Snyder, Joel St-Aubin

**Affiliations:** ^1^ Department of Biomedical Engineering, University of Iowa, Iowa City, IA, United States; ^2^ Department of MR Therapy, Philips Healthcare, Cleveland, OH, United States; ^3^ Department of Radiation Oncology, University of Iowa Health Care, Iowa City, IA, United States; ^4^ Philips Healthcare, Best, Netherlands; ^5^ Department of Therapeutic Radiology, School of Medicine, Yale University, New Haven, CT, United States

**Keywords:** MR-linac, T1-weighted cine MRI, motion monitoring, tumor tracking, T1-weighted imaging

## Abstract

**Purpose:**

High temporal resolution cine magnetic resonance imaging (MRI) for the 1.5T Elekta Unity MR-Linac system currently relies on a balanced contrast sequence for motion monitoring (MM) and tumor tracking. Despite its high signal-to-noise ratio (SNR), a balanced contrast sequence does not always provide the ideal contrast for tumor imaging in all situations. Thus, the investigation of other contrast high temporal resolution cine MRI sequences is needed.

**Methods:**

Experiments were conducted to validate the T1-weighting and SNR on a cine MRI sequence with a frame rate of 4 frames-per-second (fps) and sufficient image quality. A ModusQA Quasar MRI4D motion phantom and Alzheimer’s Disease Neuroimaging Initiative (ADNI) phantom were used to confirm adequate motion tracking, contrast-to-noise ratio (CNR), SNR, and T1 weighting of the new cine MRI sequence. Target tracking success using Elekta’s Comprehensive Motion Management (CMM) algorithm was assessed on *in vivo* patient images, and the CNR was measured for the patients with liver tumors which are one of the most challenging sites for visualization using the balanced cine MRI sequence.

**Results:**

The T1-weighted cine MRI sequence exhibited consistent CNR, SNR and T1-weighting over the duration of the scan while maintaining the ability to capture target motion within 1 mm at 4 fps. *In-vivo* analysis showed that the T1-weighted sequence had an average tracking success rate of 99.3% ± 1.12% versus the 83.3% ± 23.4% success rate of the bTFE sequence using Elekta’s CMM algorithm for all anatomical sites investigated and better CNR compared to the bTFE sequence for all liver tumors investigated.

**Conclusion:**

The proposed T1-weighted cine MRI sequence can produce quality T1-weighted images capable of tracking tumor motion over time. This demonstrates the sequence’s potential in motion monitoring tasks as an alternative to the bTFE sequence when necessary.

## 1 Introduction

Magnetic resonance guided radiation therapy (MRgRT) provides the ability to visualize the patient anatomy in exquisite detail immediately prior to treatment (1–4). It also allows for the adaptation of the treatment plan based on the current patient anatomy, tumor size, and tumor and organ at risk (OAR) locations. MRgRT provides the ability to acquire cine images during the radiation treatment to allow for real-time tumor tracking (5–9).The Elekta Unity 1.5T MR-Linac is an example of an MRgRT system that enables adaptive 7 MV flattening filter free (FFF) treatments and real-time motion monitoring (1, 10).

The ability to track the tumor in real-time enables gated radiation delivery by assessing whether the tumor is in the proper location as defined during the treatment planning process. The radiation beam is then turned on and off at appropriate times to ensure proper target coverage during its motion cycle. This in turn leads to excellent treatment responses with low toxicities (6, 11, 12). The effectiveness of treatment gating heavily depends on the quality of the real-time motion monitoring (MM) images acquired during treatment. Tumor tracking using MRI can broadly be categorized as single slice (2D) cine imaging and 3D volumetric cine imaging. 2D imaging is the current standard for tumor tracking on clinical MR-Linac systems, but 3D volumetric cine imaging is also being investigated (13, 14).

On the Elekta Unity MR-Linac system, the current clinical standard for tumor tracking is a 2D is the balanced Turbo Field Echo (bTFE) MM sequence. The bTFE sequence provides a high signal-to-noise ratio (SNR) at a high temporal frame rate of 5.6 frames-per-second (fps). However, due to its T2/T1 contrast, the target may not be visible in the bTFE sequence and may require the use of surrogate tracking structures (15). While typically better than x-ray-based imaging, liver tumors in particular may be poorly visualized, often needing a gadolinium-based contrast agent (GBCA) (16, 17) due to the large variability in imaging characteristics of hepatocellular carcinoma (HCC) nodules as well as oligometastatic disease and other metastases (11, 18). While initially there were concerns about the use of GBCA’s in conjunction with radiation treament (19, 20), further investigation suggests that this may not be an issue (21). However, contrast agents further complicate the treatment process and introduce a temporal dependence in tumor visualization as the contrast washes out. In this study, we validated a 2D T1-weighted cine MM MRI sequence that is compatible with the current Unity tumor tracking algorithm and investigated its potential to be used as an alternative to the current clinical bTFE sequence. Having access to a T1-weighted cine MRI sequence would provide clinicians with an option to improve the target visibility for tumor tracking in cases where the bTFE sequence is insufficient.

## 2 Methods

### 2.1 Sequence modification

The creation of a high temporal resolution T1-weighted cine MRI sequence entailed the adjustment of standard parameters such as the echo time (TE), repetition time (TR), and flip angle (FA) for a turbo field echo (TFE) sequence to maximize T1 weighting and SNR, while providing a high frame rate that would accurately capture tumor motion. Two phantoms were used to evaluate the resulting image quality of the sequence before *in-vivo* testing.

### 2.2 Phantom studies

#### 2.2.1 Motion phantom

**Figure 1:**
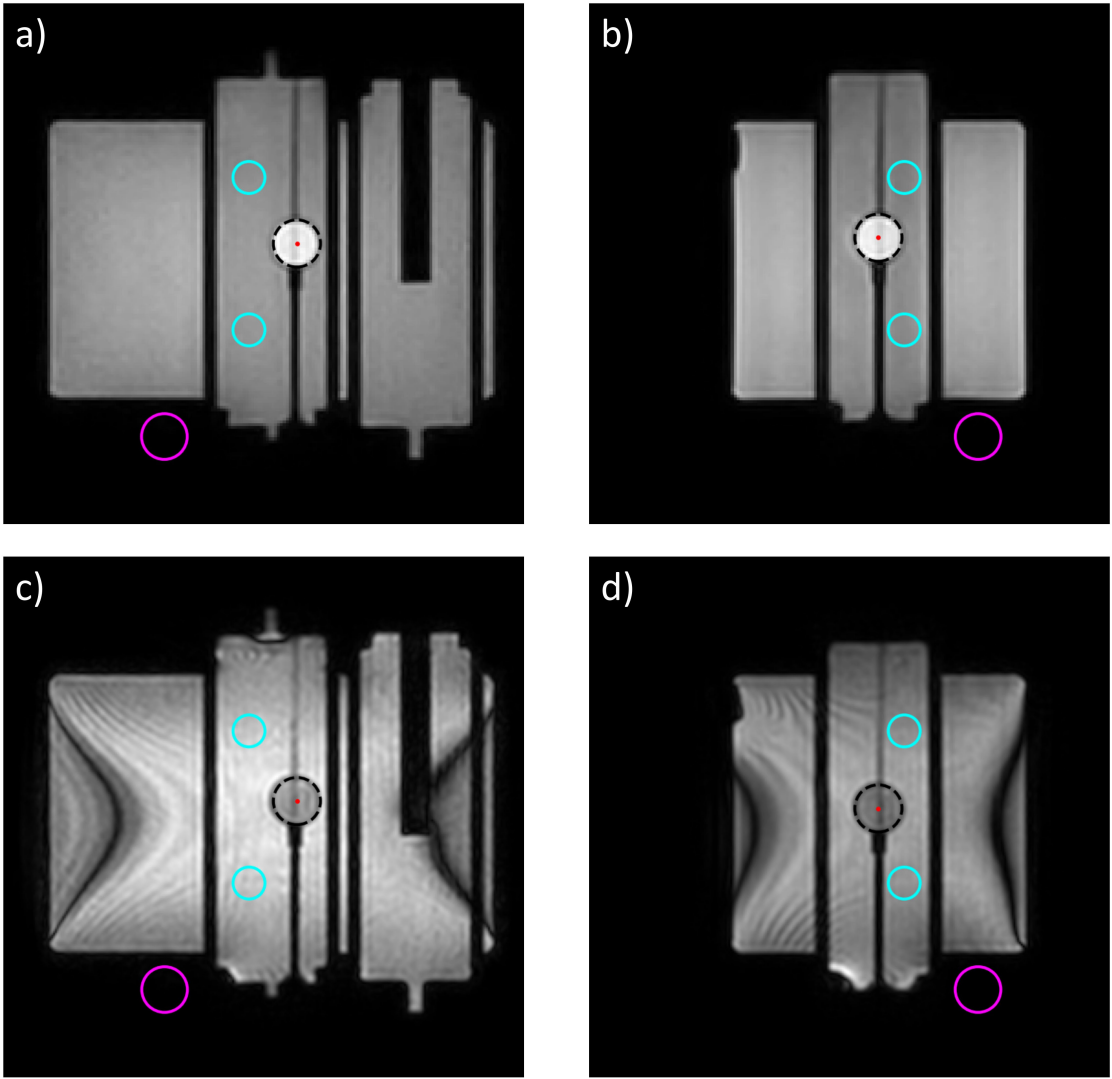
Coronal and sagittal frames of the T1-cine **(a, b)** and bTFE **(c, d)** scans with the piston ROI (cyan), air ROI (magenta), and target ROI (black dashed) shown on each scan.

The Quasar MRI4D phantom was used to evaluate the T1-weighted MRI cine scan’s ability to accurately track the motion of a simulated target. These motion experiments were also performed on the current clinical bTFE cine MRI sequence to serve as a comparison. The Quasar MRI4D phantom is composed of a main body and two pistons, one of which contains a central sphere representing the target for tracking (**Figure 1**). Differing concentrations of $MNCl_{2}\cdot 4H_{2}O$ solution is used to provide regions of differing contrast on acquired MRI images (22). Two motion patterns were chosen to simulate patient breathing. First, a sinusoidal motion of 30 mm with breathing frequencies ranging from 10–18 breaths per minute (bpm) was used to represent the range and frequency of respiratory motion that is common for many patients. Second, the target motion from a sample patient was modeled to demonstrate robustness in a real clinical scenario. A python-3.12 script was used to locate the target centroid using a Hough transform (23) and track the centroid motion of the target for the duration of the 100 sec scan. The parameters of the Hough transform were optimized for each scan until a circle of the correct radius, plus or minus half a pixel, was detected for every slice in the time series. This enabled centroids to be determined to the nearest half pixel of 0.947 mm and 0.603 mm for the T1 and bTFE cine MRI scans respectively. The mean absolute error (MAE) was then calculated between the true centroid and the centroid located from each sequence. The Hough transform was chosen as an independent method to validate image accuracy due to its ease of implementation and is different than methods used in clinical tracking algorithms.

A region of interest (ROI) was placed within the target and compared to ROIs in the air and moving piston to calculate the signal-to-noise ratio (SNR) and contrast-to-noise ratio (CNR) respectively (**Figure 1**). The formulas for CNR and SNR are shown in Equations 1, 2 respectively.


(1) $$CNR=\frac{S_{target}-S_{piston}}{\sigma_{piston}}$$

where $S_{target}$ is the average signal in the target ROI, $S_{piston}$ the average signal in the piston ROI, and $\sigma_{piston}$ the mean standard deviation over the piston ROI,


(2) $$SNR=0.66\frac{S_{target}}{\sigma_{air}}$$

with $\sigma_{air}$ being the standard deviation of the air ROI. CNR and SNR were tracked over the duration of the scan to evaluate stability over time.

#### 2.2.2 ADNI phantom

The Alzheimer’s Disease Neuroimaging Initiative (ADNI) Magphan EMR051 quantitative imaging phantom (24) (PhantomLab, Salem NY, USA) was used to quantitatively assess the ability of the proposed T1-weighted cine MRI sequence to produce images with sufficient T1 weighted contrast. The ADNI phantom contains multiple contrast spheres, each with different concentrations of copper sulfate pentahydrate. When scanned, the varying concentrations produce images with varying levels of contrast depending on the T1 weighting of the scan (24). Images of the phantom were acquired using the proposed T1-weighted cine sequence, the clinical bTFE cine sequence, and a 3D T1-weighted sequence. The T1 weighting of each scan was evaluated by taking the mean intensity ratio of the four 3.0 cm contrast spheres and the center sphere. These contrast ratios were then compared between the scans to demonstrate their relative T1 weighting and tracked for the duration of the scan (100 seconds) to show stability.

### 2.3 *In-vivo* analysis

The *in-vivo* analysis using patient data was reviewed and approved by the University of Iowa IRB (IRB00000099) under application 201109821. Prospective consent was obtained for all participants; none of the elements were waived. The study complied with ICH E6(R2) as adopted by US FDA. For the *in-vivo* study, patients received their standard of care treatment including an initial 3D MRI for adaptive planning that was determined by the treating physician, and the clinical standard bTFE cine MRI to monitor and track patient tumors during treatment delivery using Elekta’s Comprehensive Motion Management (CMM) solution. CMM provides the user with the option for respiratory gating which is ideal for tumors in the upper abdomen and thorax where respiratory motion is prominent, and exception gating for tumors that don’t move with respiration. Both CMM strategies use the same motion tracking algorithm, but respiratory gating includes a predictive algorithm to reduce the system latency.

The T1-weighted cine MRI sequence was acquired outside of the clinical workflow with the patient’s prospective consent. 400 frames (100 sec) of T1-weighted cine MRI data were acquired and analyzed for a total of 11 patients to assess the temporal resolution and image quality of the T1-weighted cine MRI scan. The T1-weighted and bTFE cine MRI data were analyzed offline in the Elekta Motion Management Research Platform (MMRP) which is a research platform that simulates the clinical CMM. With the MMRP, the ability of the target tracking algorithm to track the target in both the bTFE and T1-weighted cine MRI sequences was compared. Elekta’s real-time target tracking algorithm relies on 2D image registration between a masked region surrounding the target on the 2D cine images and a template set of 2D images extracted from the 3D volume. During this registration process the system evaluates whether any of the following problematic situations occur: Large anatomy deformations, through-plan motion, or jitter. The detection of any one of the above issues constitutes a failure in tracking and treatment would be prevented. These metrics were designed to prevent treatment with cases of poor image quality, insufficient contrast, and deformations.

For each imaging frame, the MMRP software returns a status of ‘success’, ‘failure’, or ‘not ready’ if the algorithm re-enters a preparation phase. Specifically, tracking success percentage after the first initialization (60 frames) was recorded and used to compare the algorithm tracking quality between the bTFE and T1-weighted cine MRI. For consistency across scans, equivalent time intervals were chosen for tracking as the bTFE sequence was acquired during treatment and run for much longer. CNR was calculated from an arbitrary 10 second window for each of the five liver cancer patients in the *in-vivo* study using an ROI located inside the tumor contour and an equivalently sized ROI in the surrounding liver tissue. The liver patients were chosen for this analysis because they are notoriously difficult to track using the bTFE sequence and had large enough tumors to reliably sample both target and liver regions for the CNR measurement.

## 3 Results

### 3.1 Sequence modification

The scan parameters for a traditional 3D T1-weighted scanT1-weighted cine MRI, and bTFE cine MRI scans are given in **Table 1**. The 3D T1-weighted acquisition was used to assess the T1-weighting of the presented T1-weighted cine MRI. The echo time for the T1-weighted cine MRI was specifically chosen as out-of-phase for an easily visualizable liver-fat boundary which is ideal for tracking. Image reconstruction time for the T1-weighted cine sequence could not be directly quantified with the tools provided on the clinical Unity MR-Linac MRI, but based on internal testing at Philips (Best, Netherlands) a compressed SENSE acceleration only added a few milliseconds to the reconstruction time compared to standard SENSE acceleration. It is expected that the use of compressed SENSE for the T1-weighted sequence will not significantly impact on the overall latency of the real-time imaging compared to the current bTFE sequence.

**Table 1:** Sequence parameters for a standard 3D T1-weighted MRI, the T1-weighted cine MRI, and current clinical bTFE cine MRI scans are given.

Sequence Parameter	3D T1	T1-weighted cine	bTFE cine
Echo Time (ms)	4.000	2.098	1.706
Repetition Time (ms)	6.162	4.900	3.413
Flip Angle (degrees)	10	15	40
Slice thickness (mm)	4.0	6.0	5.0
In-Plane Resolution (mm)	0.989	1.893	1.205
Water-fat shift	0.482	0.435	0.201
Frame rate (frames-per-second)	N/A	4.00	5.56
Acceleration	Compressed SENSE= 2.12	Compressed SENSE= 3.0	SENSE = 3.0

N/A, not applicable.

#### 3.1.1 Motion phantom studies

**Figure 2:**
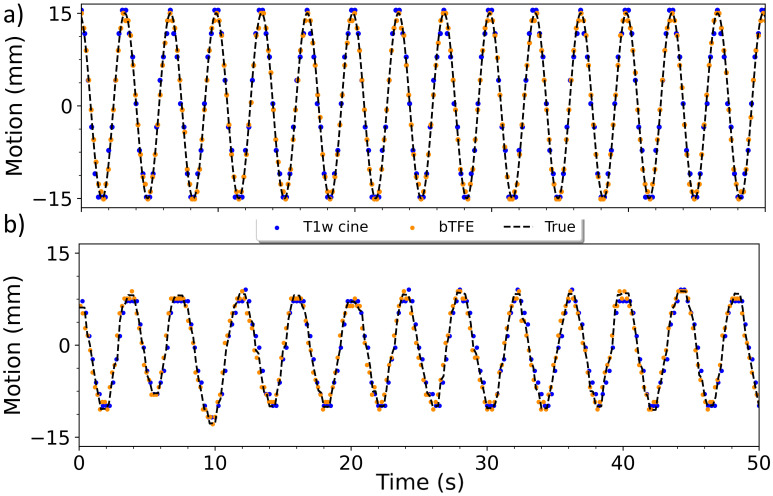
Plot of the target centroid over 50 seconds for the bTFE and the T1-cine scans showing the sinusoidal motion at 18 bpm **(a)** and the simulated patient motion **(b)**.

**Figure 3:**
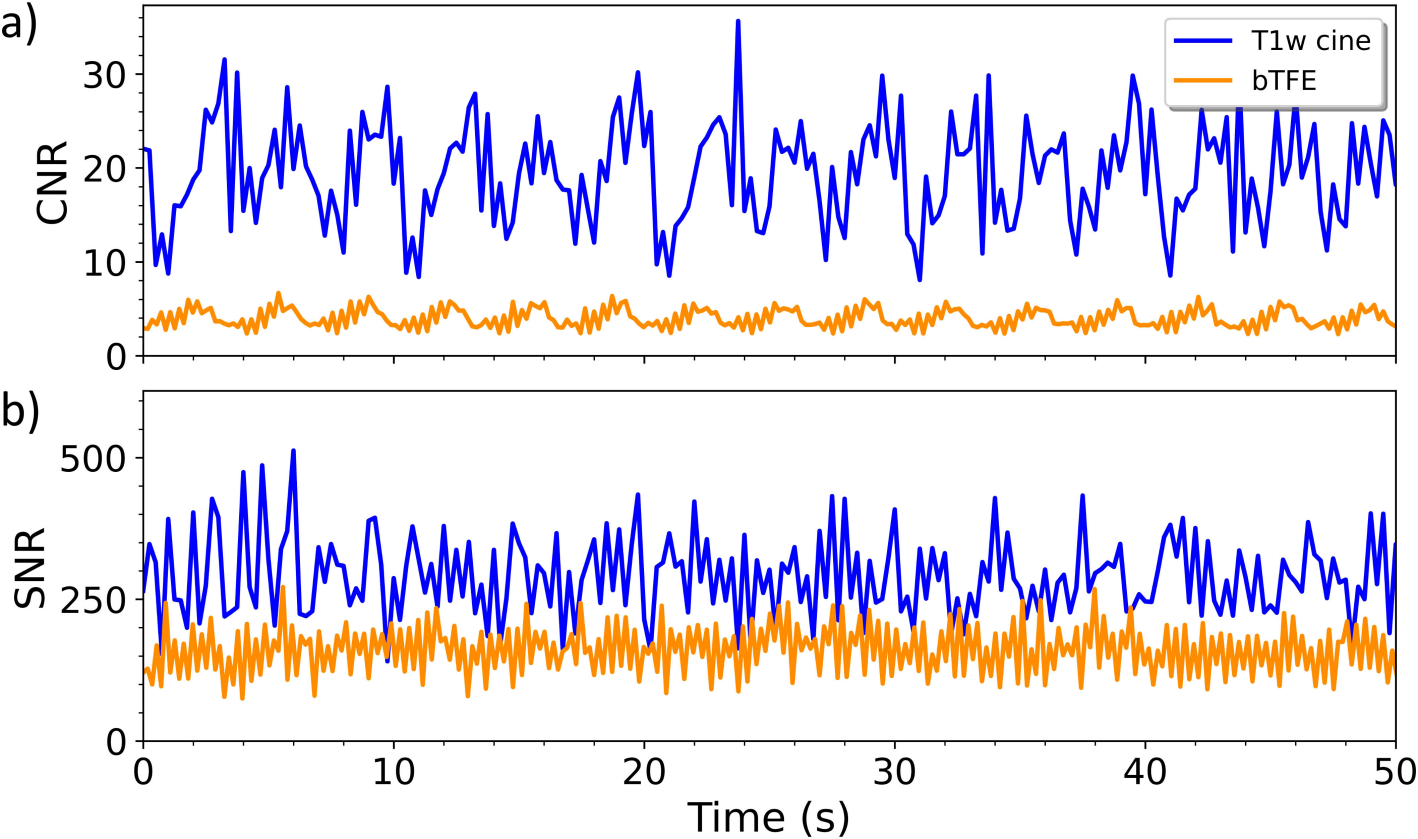
Plot of the The CNR **(a)** and SNR **(b)** at a frequency of 18 bpm over 50 seconds for the bTFE and the T1-cine scan.

For the 30 mm sinusoidal motion pattern, the MAE between the true and located centroid motion was 0.83 ± 0.64 mm for the T1 weighted scan, and 0.77 ± 0.67 mm for the bTFE scan. The MAE of the centroid locations from the simulated patient motion was 0.95 ± 0.63 mm and 0.80 ± 0.60 mm for the T1 weighted and bTFE sequences respectively. A 50 second interval of the motion from each breathing pattern can be seen in **Figure 2**. The T1-weighted cine scan had a higher mean CNR of 19.51 ± 5.58 compared to the 3.86 ± 0.97 of the bTFE scan. Similarly, it had a higher mean SNR of 289.88 ± 72.34 compared to the 160.74 ± 43.54 of the bTFE scan. **Figure 3** shows plots of CNR and SNR for both sequences at 18 bpm, the highest evaluated breathing frequency.

#### 3.1.2 ADNI phantom

**Figure 4:**
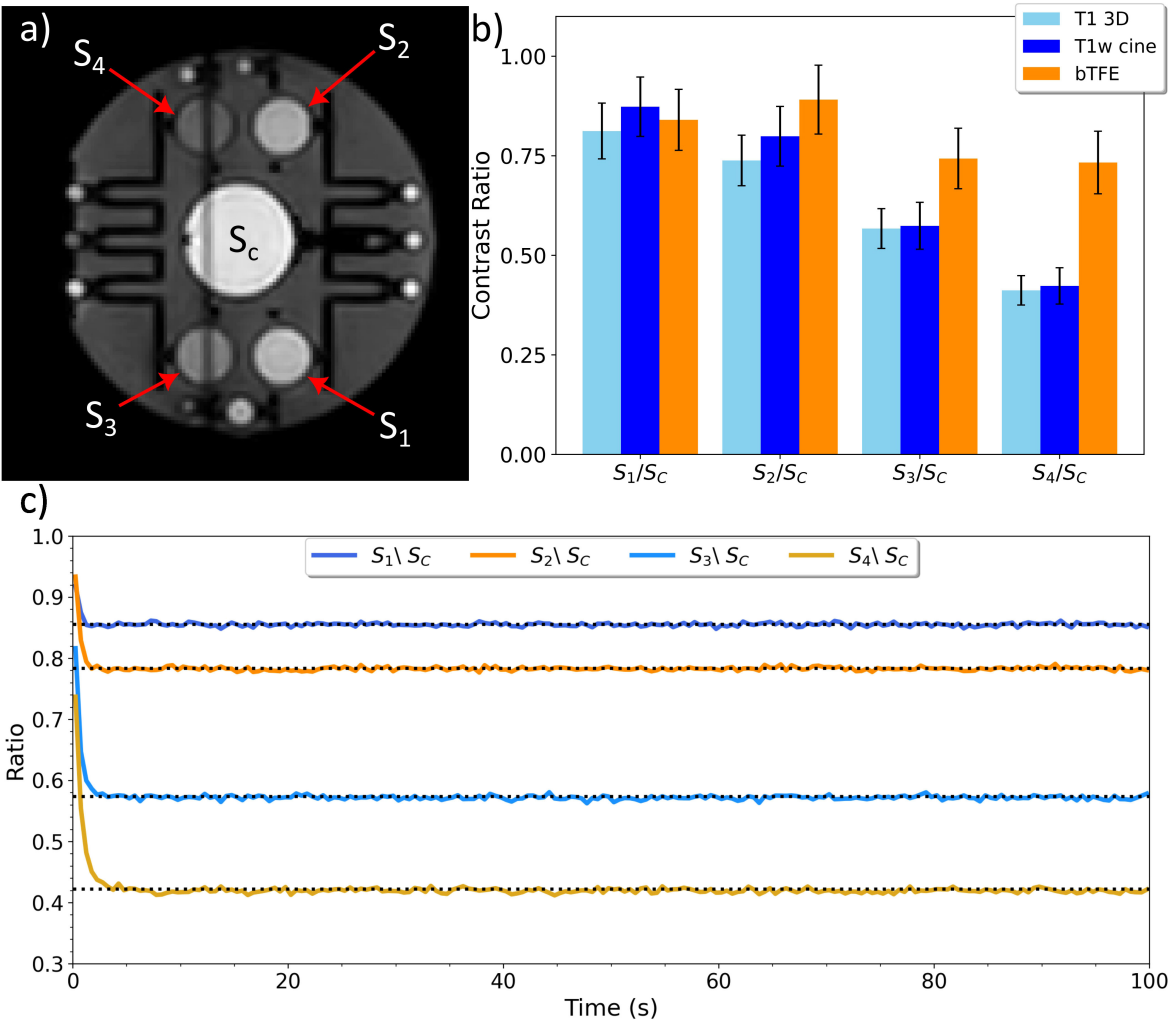
A sagittal frame of the ADNI phantom for the T1-weighted cine MRI sequence **(a)**, a plot showing the contrast sphere intensity ratios for a clinical 3D T1 scan, the T1-weighted cine scan, and the clinical bTFE scan **(b)**, and a plot of the intensity ratios from each constrast sphere for every frame of the T1-weighted cine MR scan **(c)**.

A T1-weighted cine sagittal frame of the ADNI phantom with the four labeled contrast spheres and the large central sphere can be seen in **Figure 4a**. **Figure 4b** shows the intensity ratios between each of the contrast spheres (S_1_, S_2_, S_3_, S_4_) relative to the central sphere (S_c_) for each scan. Calculations for spheres S3 and S4 were performed excluding the crosstalk band. The contrast ratios for the T1-weighted cine scan closely resembled the T1 3D scan, suggesting that the developed sequence is sufficiently T1-weighted. The intensity ratios at each time point were plotted in **Figure 4c**. The ratios for the proposed scan remain consistent over the duration of the scan after initially reaching equilibrium. Quantitative results of the intensity ratios of the T1-weighted cine MRI on the ADNI phantom are given in **Table 2**.

**Table 2:** Intensity ratios between each of the four contrast spheres and the larger central sphere for a 3D T1-weighted scan, the developed T1-weighted cine MRI scan, and bTFE cine MRI scan.

Scan	$S_{1}/S_{C}$	$S_{2}/S_{C}$	$S_{3}/S_{C}$	$S_{4}/S_{C}$
T1 3D	0.812 ± 0.140	0.738 ± 0.127	0.567 ± 0.100	0.412 ± 0.074
T1-w cine MRI	0.873 ± 0.149	0.799 ± 0.150	0.574 ± 0.118	0.423 ± 0.091
bTFE cine MRI	0.840 ± 0.153	0.891 ± 0.173	0.743 ± 0.152	0.733 ± 0.157

### 3.2 *In-vivo* analysis

The treatment site, primary disease information, and tracking success rate are shown for each of the 11 imaged patients in **Table 3**. These results suggest that the T1-weighted cine MRI sequence provided more reliable tracking using the Elekta CMM algorithm. Clinically, patient 4 required the use of a surrogate structure (15) to achieve reliable tracking. The image planes are taken at the centroid of the tracking structure (surrogate) and in this case the sagittal plane did not pass through the target. Thus, we were unable to assess the tracking success of the bTFE sequence for patient 4. The improvement in tracking for the T1-weighted cine MRI on the 10 patients (excluding patient 4) was statistically significant using a single tailed t-test ( p=0.028) .

**Table 3:** The treatment site, treated disease, and tracking success rate each of the 11 patients is provided. The measured CNR is also listed for the 5 liver patients.

Target Tracking Assessment				
Patient	Site	Treated Disease	Imaging Sequence	Tracking Success %
1	Liver	Metastasis	T1	100.0
			bTFE	69.5
2	Liver	Metastasis	T1	100.0
			bTFE	68.7
3	Lung	Squamous Cell Carcinoma	T1	97.3
			bTFE	21.0
4	Liver	Cholangiocarcinoma	T1	100.0
			bTFE	N/A
5	Abdomen	Leiomyosarcoma	T1	100.0
			bTFE	96.3
6	Head + Neck	Squamous Cell Carcinoma	T1	100.0
			bTFE	92.6
7	Liver	Metastatic Choroidal Melanoma	T1	100.0
			bTFE	96.2
8	Kidney	Renal Cell Carcinoma	T1	97.0
			bTFE	96.8
9	Liver	Metastatic Pancreatic Cancer	T1	100.0
			bTFE	95.2
10	Abdomen	Intra-abdominal Follicular Lymphoma	T1	100.0
			bTFE	98.7
11	Adrenal	Metastasis	T1	98.5
			bTFE	97.7
CNR Assessment				
Patient	Site	Treated Disease	Imaging Sequence	CNR
1	Liver	Metastasis	T1	9.12 ± 4.37
			bTFE	1.26 ± 1.33
2	Liver	Metastasis	T1	8.36 ± 3.82
			bTFE	3.62 ± 2.23
4	Liver	Cholangiocarcinoma	T1	4.33 ± 1.97
			bTFE	2.49 ± 2.11
7	Liver	Metastatic Choroidal Melanoma	T1	5.14 ± 3.23
			bTFE	2.63 ± 0.96
9	Liver	Metastatic Pancreatic Cancer	T1	4.50 ± 3.11
			bTFE	2.77 ± 1.72

**Figure 5:**
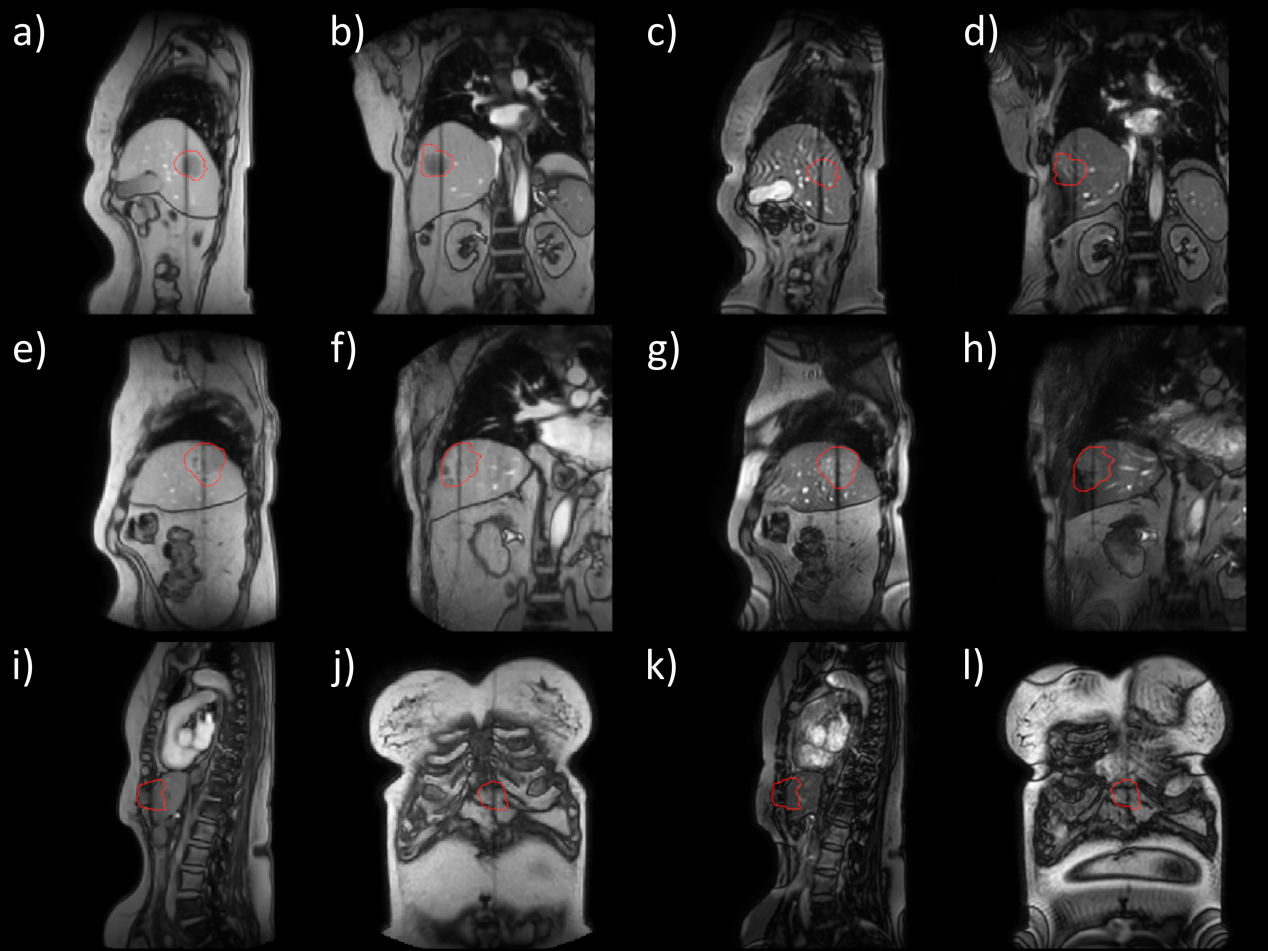
2D cine images captured mid sequence acquisition for liver tumors treated on the Unity for patient 1 **(a-d)**, patient 7 **(e-h)**, and patient 9 **(i-l)**. The shown images are T1-weighted cine MRI sagittal **(a, e, i)** and coronal **(b, f, j)**, bTFE cine MRI sagittal **(c, g, k)** and coronal **(d, h, l)**. The target is lightly outlined in red.

In all liver cancer patients, the calculated CNR was higher for the T1-weighted cine MRI sequence compared to the bTFE. The highest difference was seen in patient 1, and this can be seen clearly in **Figures 5a-d**. Patients 7 (**Figures 5e-h**) and 9 (**Figures 5i-l**) are also shown as examples of liver tumors with lower CNR. Figures of the other patients imaged in this study are in the Supplementary document.

## 4 Discussion

The T1-weighted and bTFE cine MRI sequences were able to accurately capture the phantom sinusoidal motion within 1 mm of the true motion across all examined breathing frequencies. Similarly, each scan was able to capture the simulated patient motion, with both scans having a mean absolute error less than 1 mm. Due to the limitations imposed by the Hough transformation and image resolution, the accuracy of the centroid position is determined to be within half a pixel (0.95 mm and 0.60 mm for the T1-weighted and bTFE cine MRI scans respectively). Consequently, the measured motion represents the closest approximation achievable through this method. While both scans were able to accurately capture the motion at these frequencies, the bTFE scan’s higher frame rate and resolution is likely the reason for achieving slightly better accuracy in centroid position and may result in better delineation of piston motion peaks and troughs at even higher breathing rates. In particular, the number of motion samples near the apex of the motion helps confirm the entire range of motion is being captured. However, even at the reduced frame rate of 4 fps, the T1-weighted scan was able to accurately capture the target motion at high breathing rates.

The CNR and SNR measured on the Modus motion phantom for the bTFE sequence could be affected by the artifacts in the image. However, the location of the ROIs were chosen to minimize this effect. Quantitatively T1-weighted cine MRI outperformed the bTFE sequence for this phantom test. However, in order to achieve good SNR for the T1-weighted cine MRI scan at the 4 fps temporal resolution, a larger in-plane pixel size and slice thickness was required. Importantly, the plot of the CNR and SNR of each scan over time shown in **Figure 3** demonstrates that the T1-weighted cine MRI scan is stable over the duration of the scan which is key when used clinically to track tumor motion over an extended treatment period.

The intensity ratios of the T1-weighted cine MRI scan showed the same contrast trend as the 3D T1-weighted scan on the ADNI phantom which indicates a similar T1 weighting. All contrast spheres demonstrated higher ratios than those observed in 3D T1-weighted scan, with spheres 3 and 4 being the closest relative to the 3D scan. Overall, based on the results of the contrast ratios, there appears to be slightly less T1 contrast for the cine MRI scan compared to the 3D T1-weighted scan due to the requirement to maintain a high frame rate for motion tracking. As expected, the bTFE cine MRI scan showed a larger discrepancy between its computed contrast ratios and those of the 3D T1-weighted scan since the bTFE sequence achieves a T2/T1 contrast. In **Figure 3c**, it can be observed that there is consistency in sphere intensity ratios over time and thus affirming the stability of the T1-weighting over time. This stability, in conjunction with the contrast weighting, demonstrates the T1-weighted cine MRI scan’s potential as an alternative to the bTFE sequence when a T1-weighting may be beneficial. This suggests the T1-weighted cine MRI scan’s potential viability as an alternative to the bTFE sequence when a T1-weighting may be beneficial.

As shown in **Table 3**, the Elekta MMRP target tracking algorithm showed notable improvement or non-inferiority in tracking success in all *in-vivo* cases for the T1-weighted cine MRI scan. Additionally, the T1-weighted scan showed improved CNR *in-vivo* for all liver patients. Obtaining images with good target contrast can be difficult in liver patients (11), and often a surrogate structure is used for motion monitoring for this reason (15, 25, 26) Using a T1 sequence to track the target directly during treatment could help minimize the error between true and tracked motion and avoid the reliance on a surrogate structure. The results of the *in-vivo* study for target tracking and CNR confirm the results shown in the phantom analysis that the T1-weighted cine MRI sequence provides a significant improvement in T1-weighting, improved CNR for the liver cancer cases studied, and better tracking success without the need for a surrogate for tracking. This success with the Elekta MMRP algorithm proves its compatibility with the Elekta prediction algorithm used to minimize latency between image acquisition and beam on trigger. Such algorithms are particularly useful in the progression of other future adaptive MRIgRT practices such as MLC tracking. The proposed T1-weighted cine MRI approach demonstrates the capability to acquire 2D images rapidly, making it suitable for current real-time motion tracking methods during treatment. However, a key limitation of this method, as with all MR-Linac systems, is that 2D images cannot completely capture 3D deformations or out-of-plane motion. While using two orthogonal 2D planes is able to significantly mitigate these errors, 3D volumetric imaging techniques need to be developed that can capture complex 3D motion and deformations with minimal latency. Approaches for 3D volumetric cine imaging include techniques such as MRSIGMA and MR-MOTUS (13, 14). These techniques rely on matching a real-time acquired signal to a previously acquired signal to estimate motion. This allows for minimal k-space acquisition enabling low-latency 3D MRI images. One potential problem with these methods, is that the under sampling required to achieve high temporal resolution can compromise spatial resolution and image contrast (27). However, these 3D cine techniques, paired with a wider selection of sequences with varying contrast mechanisms, could significantly improve target tracking during radiotherapy treatment on MR-Linacs.

Another limitation of this study is the duration in which the T1-weighted cine MRI was acquired. The acquisition of 100 sec of data for the T1-weighted cine MRI was a balance between acquiring sufficient data for a stability analysis and not substantially extending the patient treatments. Standard treatment appointments for the patients in this study were generally between 25–60 min depending on treatment site and complexity. A more appropriate assessment of tracking success would be to acquire data of a duration similar to the standard CMM tracking time of 10–15 min, which was not feasible for the *in-vivo* study in this work. However, stability of the CNR and SNR was shown within the 100 sec acquisition, so the additional scan time needed for treatment is not expected to change the conclusions of this work. Another limitation is that a T1-weighted cine MRI may not be appropriate for every clinical case, and it is up to the clinical team’s judgement on which sequence to use. Although the sample size of the *in-vivo* study was small, a sampling of many representative treatment sites is shown. However, despite the small sample size, for all cases investigated the proposed T1-weighted cine MRI scan has demonstrated equal or improved tumor visibility *in-vivo* and improved tumor tracking success for the Elekta CMM solution. Having a high frame rate T1-weighted cine MRI can as an option is expected to improve the contrast and tracking accuracy in many cases including liver treatments.

## 5 Conclusions

In this study we validated a T1-weighted cine sequence with a sufficient resolution and frame rate to be used as an additional option for motion monitoring during radiotherapy treatment. In a motion phantom, the proposed T1-weighted cine MRI sequence showed improved CNR and SNR compared to the bTFE cine MRI sequence while maintaining the frame rate necessary to capture the target motion within 1 mm. In the ADNI contrast phantom, the T1-weighted cine sequence showed similar relative T1 weighted contrast to the 3D T1-weighted scan that remained stable over the scan duration. *In-vivo*, the T1-weighted cine MRI had a similar or higher rate of target tracking success, with the worst case showing a success rate of 97.0%. Additionally, the CNR for the *in-vivo* T1-weighted cine MRI patient imaging was higher for all selected liver patients compared to the bTFE cine MRI with the greatest difference of 9.12 ± 4.37 and 1.26 ± 1.33 respectively. Overall, these findings demonstrate that the proposed T1-weighted cine MRI sequence has strong potential for use in motion monitoring tasks and can be used as an alternative to the bTFE sequence when appropriate.

## Data Availability

The datasets presented in this article are not readily available because of protected and/or private heath information. Requests to access the datasets should be directed to the corresponding author (Joel St-Aubin, joel-st-aubin@uiowa.edu).

## Supplementary material

Supplementary Figure 1 Images from patient 2. The shown images are T1-weighted cine MRI sagittal **(a)** and coronal **(b)**, bTFE cine MRI sagittal **(c)** and coronal **(d)**. The target is lightly outlined in red.

Supplementary Figure 2 Images from patient 3. The shown images are T1-weighted cine MRI sagittal **(a)** and coronal **(b)**, bTFE cine MRI sagittal **(c)** and coronal **(d)**. The target is lightly outlined in red.

Supplementary Figure 3 Images from patient 4. The shown images are T1-weighted cine MRI sagittal **(a)** and coronal **(b)**, bTFE cine MRI sagittal **(c)** and coronal **(d)**. The target is lightly outlined in red.

Supplementary Figure 4 Images from patient 5. The shown images are T1-weighted cine MRI sagittal **(a)** and coronal **(b)**, bTFE cine MRI sagittal **(c)** and coronal **(d)**. The target is lightly outlined in red.

Supplementary Figure 5 Images from patient 6. The shown images are T1-weighted cine MRI sagittal **(a)** and coronal **(b)**, bTFE cine MRI sagittal **(c)** and coronal **(d)**. The target is lightly outlined in red.

Supplementary Figure 6 Images from patient 8. The shown images are T1-weighted cine MRI sagittal **(a)** and coronal **(b)**, bTFE cine MRI sagittal **(c)** and coronal **(d)**. The target is lightly outlined in red.

Supplementary Figure 7 Images from patient 10. The shown images are T1-weighted cine MRI sagittal **(a)** and coronal **(b)**, bTFE cine MRI sagittal **(c)** and coronal **(d)**. The target is lightly outlined in red.

Supplementary Figure 8 Images from patient 11. The shown images are T1-weighted cine MRI sagittal **(a)** and coronal **(b)**, bTFE cine MRI sagittal **(c)** and coronal **(d)**. The target is lightly outlined in red.
