# Correction: Validating a T1-weighted cine MRI for a 1.5T MR-Linac with temporal resolution appropriate for respiratory motion

**DOI:** 10.3389/fonc.2025.1682399

**Published:** 2025-10-23

**Authors:** Nathan Shaffer, Alex Dresner, Qi Ying, Eveline Alberts, Marijn Kruiskamp, Joseph Caster, Daniel Hyer, Jeffrey Snyder, Joel St-Aubin

**Affiliations:** ^1^ Department of Biomedical Engineering, University of Iowa, Iowa City, IA, United States; ^2^ Department of MR Therapy, Philips Healthcare, Cleveland, OH, United States; ^3^ Department of Radiation Oncology, University of Iowa Health Care, Iowa City, IA, United States; ^4^ Philips Healthcare, Best, Netherlands; ^5^ Department of Therapeutic Radiology, School of Medicine, Yale University, New Haven, CT, United States

**Keywords:** MR-linac, T1-weighted cine MRI, motion monitoring, tumor tracking, T1-weighted imaging

**Supplementary Figures 1–8** were omitted. The files have now been published.

The original article has been updated.

