# Supplementary figures and images for: Validating a T1-weighted cine MRI for a 1.5T MR-Linac with temporal resolution appropriate for respiratory motion
Source: Front Oncol. 2025 Jun 4;15:1575001. doi: 10.3389/fonc.2025.1575001 (PMC12174060; doi:10.3389/fonc.2025.1575001)

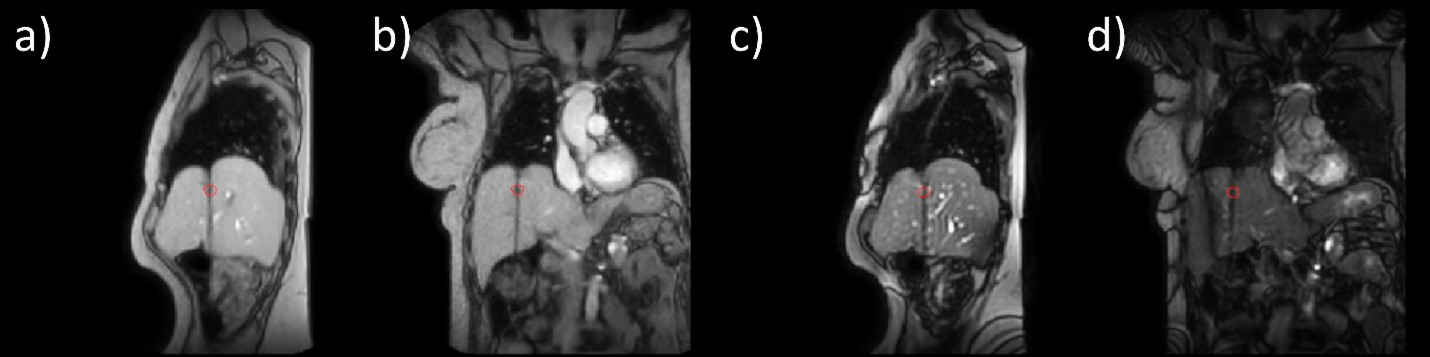

Supplement: Supplementary Figure 1 — Images from patient 2. The shown images are T1-weighted cine MRI sagittal (a) and coronal (b), bTFE cine MRI sagittal (c) and coronal (d). The target is lightly outlined in red. [file Image1.jpeg]

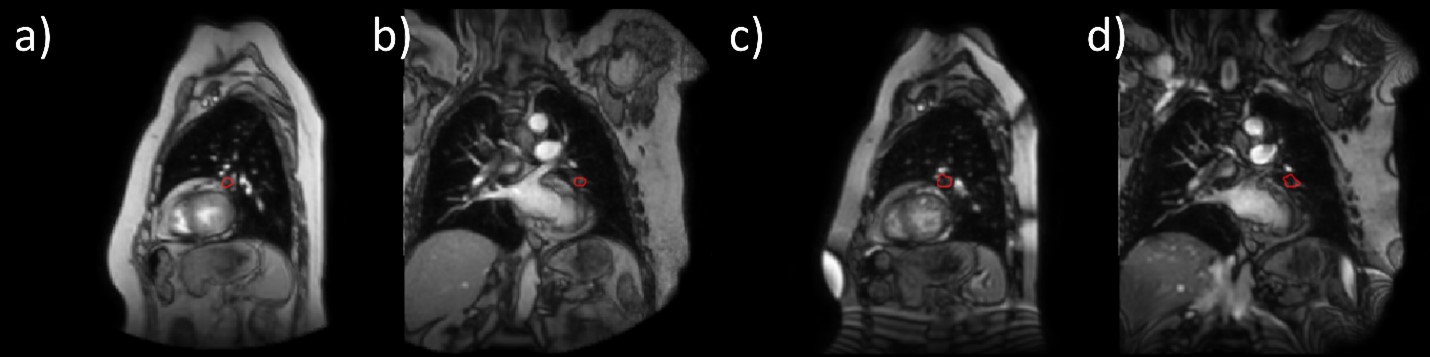

Supplement: Supplementary Figure 2 — Images from patient 3. The shown images are T1-weighted cine MRI sagittal (a) and coronal (b), bTFE cine MRI sagittal (c) and coronal (d). The target is lightly outlined in red. [file Image2.jpeg]

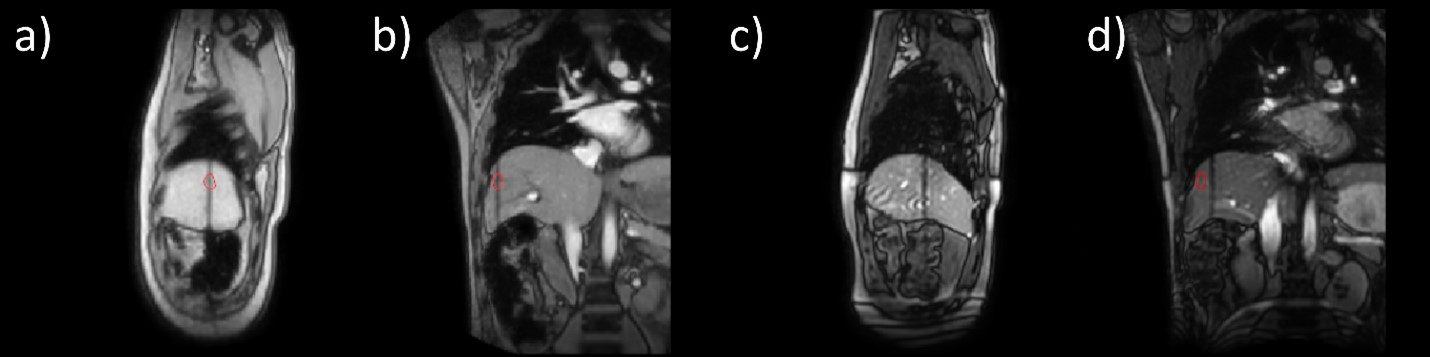

Supplement: Supplementary Figure 3 — Images from patient 4. The shown images are T1-weighted cine MRI sagittal (a) and coronal (b), bTFE cine MRI sagittal (c) and coronal (d). The target is lightly outlined in red. [file Image3.jpeg]

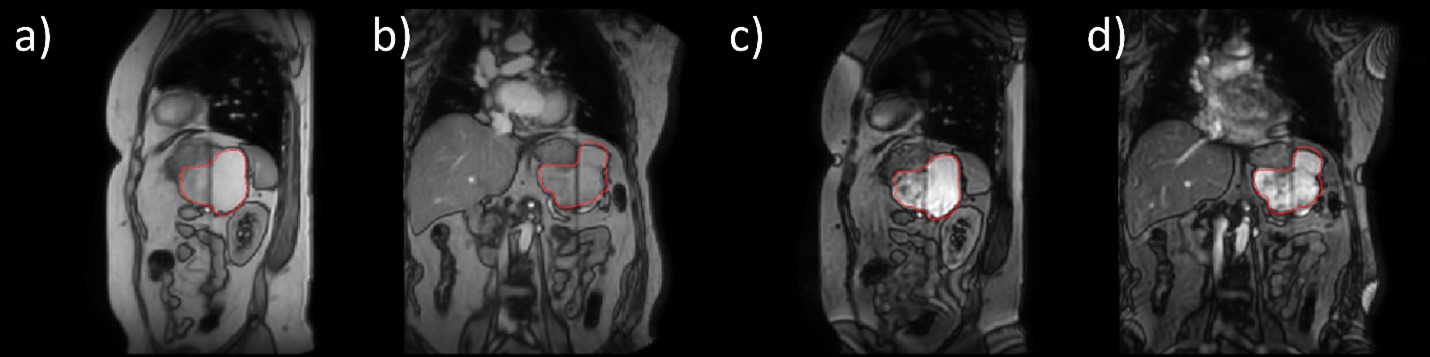

Supplement: Supplementary Figure 4 — Images from patient 5. The shown images are T1-weighted cine MRI sagittal (a) and coronal (b), bTFE cine MRI sagittal (c) and coronal (d). The target is lightly outlined in red. [file Image4.jpeg]

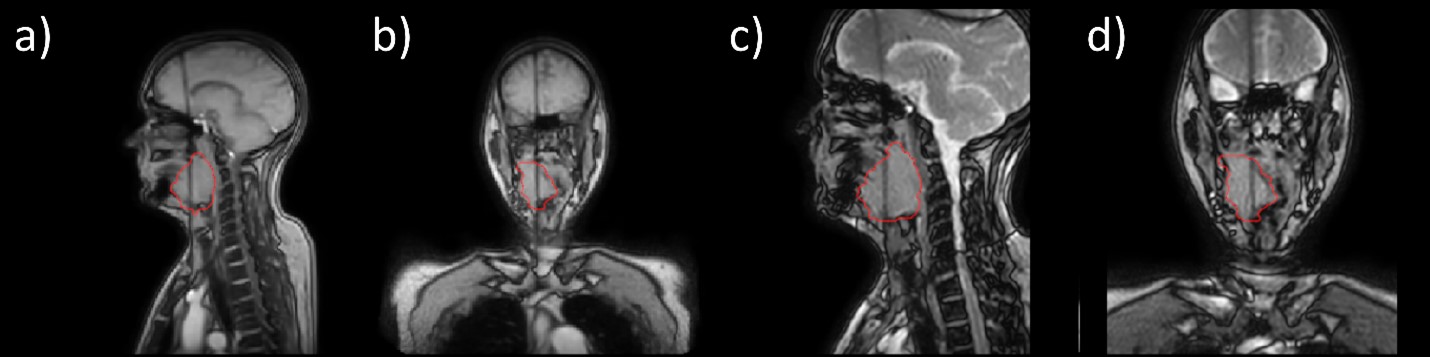

Supplement: Supplementary Figure 5 — Images from patient 6. The shown images are T1-weighted cine MRI sagittal (a) and coronal (b), bTFE cine MRI sagittal (c) and coronal (d). The target is lightly outlined in red. [file Image5.jpeg]

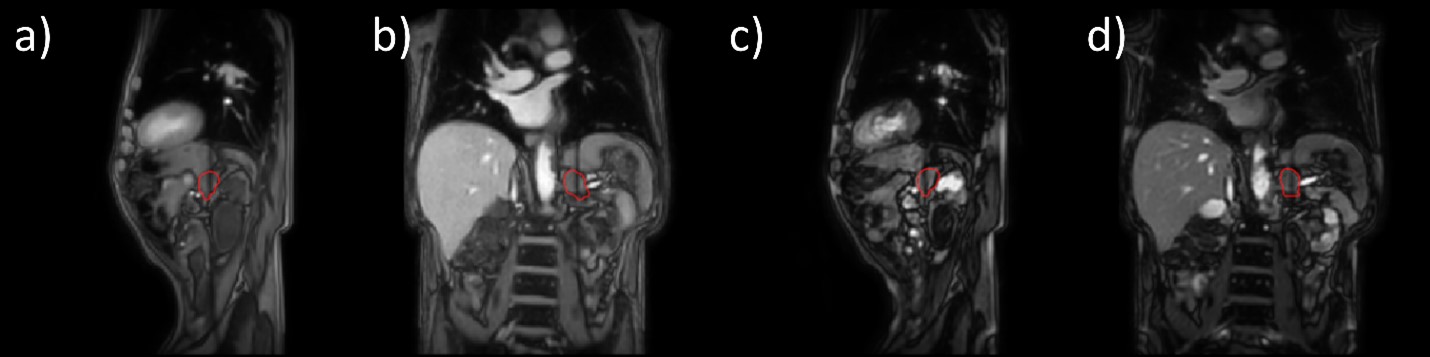

Supplement: Supplementary Figure 6 — Images from patient 8. The shown images are T1-weighted cine MRI sagittal (a) and coronal (b), bTFE cine MRI sagittal (c) and coronal (d). The target is lightly outlined in red. [file Image6.jpeg]

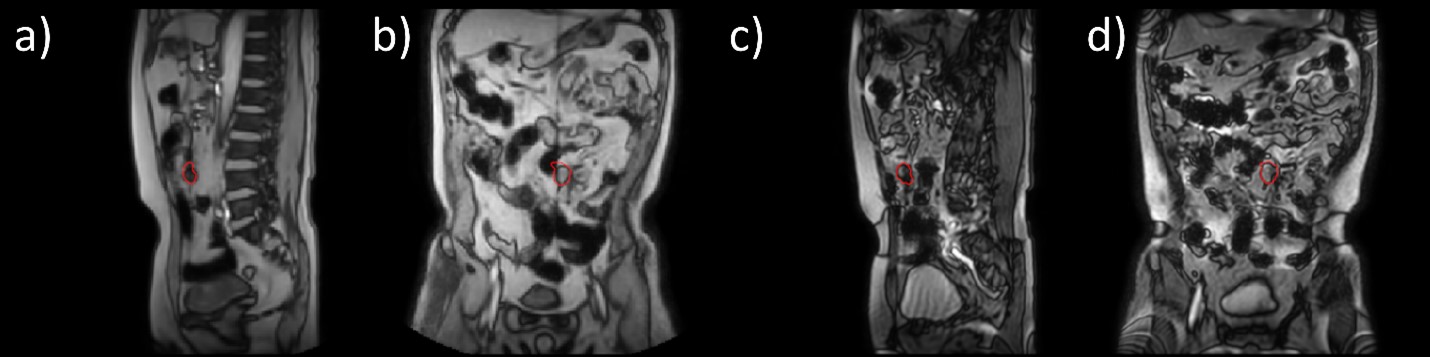

Supplement: Supplementary Figure 7 — Images from patient 10. The shown images are T1-weighted cine MRI sagittal (a) and coronal (b), bTFE cine MRI sagittal (c) and coronal (d). The target is lightly outlined in red. [file Image7.jpeg]

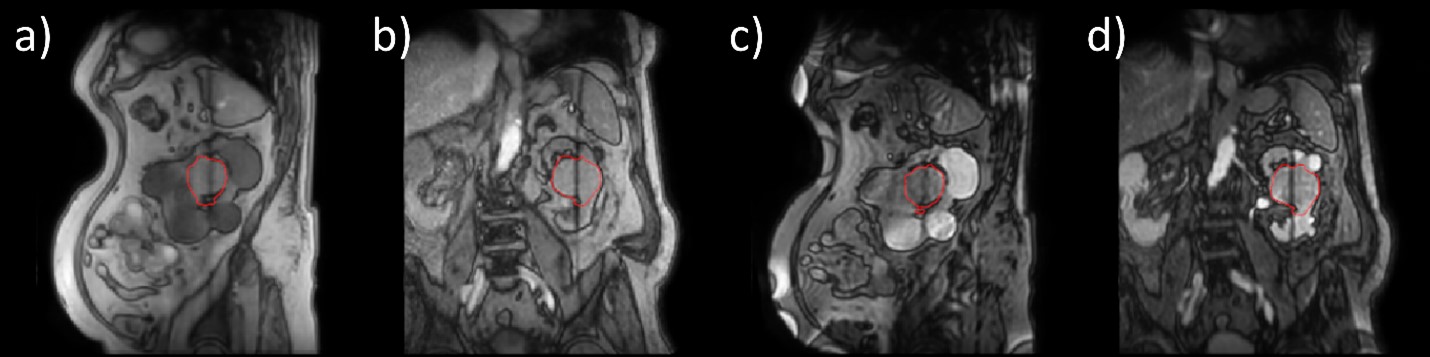

Supplement: Supplementary Figure 8 — Images from patient 11. The shown images are T1-weighted cine MRI sagittal (a) and coronal (b), bTFE cine MRI sagittal (c) and coronal (d). The target is lightly outlined in red. [file Image8.jpeg]
